# Supplementary material for: Bon bagay (good stuff): A faith-based outlook on biomedical prevention among Haitians and Haitian Americans
Source: PLoS One. 2025 Aug 25;20(8):e0330852. doi: 10.1371/journal.pone.0330852 (PMC12377581; doi:10.1371/journal.pone.0330852)
Supplement: S1 Appendix — (DOCX) [file pone.0330852.s001.docx]

**Appendix**

**Qualitative Focus Group Guide for Church Leaders**

| **Topics** | **Questions** | **Quotes** |
| --- | --- | --- |
| **Structure of church/objectives** | - How is your church organized? - How are decisions made at the church? - What are the overall goals of the church? | *"It’s the church’s committee that represents the majority... that decision stands and the church will follow."*  *“Um, well, the goal of the church mostly is, um, to connect, serve, and grow.”*  *“You mentioned it, our objective now is discipleship. We want to grow in number, we want to educate our followers. That is our main goal. Grow spiritually.”* |
| **Thoughts regarding HIV/AIDS** | - Do people in the community talk about HIV? - When you think of HIV what are your first thoughts/ what comes to mind? - What are your perceptions of HIV in the Haitian community in Miami? | *"Sometimes I am under the impression our society forgot that AIDS exists."*  *“It is just not spoken about in the Haitian community… they're too ashamed to speak about it.”*  *“It’s a subject that’s basically taboo because we know of AIDS, but many people don’t talk about it or comment about it…”*  *“A monster, because uh, growing up that’s what I used to hear. It’s a monster.”*  *“Alright, so HIV is a very sensitive subject. Mm-hmm. So, this is not something that people openly discuss. So, it's just like, that's like the family, what the skeletons in the closet basically.”*  *"We sometimes hear about infected people pricking their fingers and dipping it into food that they are preparing or in juices that others will drink."*  *“I would say the stigma still exists even among professionals. Because even sometimes at work, let’s say you gave a report to someone of a patient, and you say this person has High5 and its like ouu... I don’t want to take this. ”*  *“We are all afraid of AIDS. All of us Haitians we are afraid of AIDS. I am not going to lie to anyone, we as Haitians once we hear someone has it, we are all afraid.”*  *“As Haitians, we can say that we are victimized by HIV because at first, they said that Haitians were the initial bearers of the disease."*  *"Haitians were the initial bearers of the disease. This caused the marginalization of Haitians."*  *“Someone who is at church and catches this disease in these kinds of activities are not honest. They are not serious. They have no respect for themselves and for God.”*  *“Some Haitians think, we can’t ignore this, AIDS is a potion that’s given to someone. Excuse me, I am explaining all this in Creole. It’s an AIDS potion…we don’t have the knowledge to know “Hey, you got it somewhere”. The disease exists, you can’t ignore it.”* |
| **Thoughts regarding PrEP** | - Have you heard of PrEP (Truvada, Descovy, and Apretude) for HIV prevention? - What are your thoughts or beliefs about PrEP for HIV prevention? - Do you think PrEP could benefit the Haitian community? | *“I've never heard of PrEP. What*  *is PrEP?”*  *“I heard about the PrEP medication. It like a person who has a mistress lifestyle, such as they are with more than one person at the time. They don’t have the disease, but they take the medication in case they have sexual relations with someone with the disease, you know, they are protecting.”*  *“It's actually a very, very good thing. Um, my only, uh, a few things that I would like to mention is the fact that, um, It, it doesn't have to go all the way that you have to take a pill for it.”*  *“You know but, I definitely, I don’t know, this Prep prevention I am still in…Yes, I think it’s a great thing I think it’s awesome but there’s other ways as believers.”*  *“This medication is helping or encouraging people to live a certain lifestyle.”*  *“Um, I think it doesn't have to go to the extent where we have to have them drinking a pill. The reason why I'm saying that is because while you've seen all of this, uh, advertisement about, um, pills, but we also know yes, it's going to prevent that, but there are so many side effects mm-hmm. To it.”*  *“You can take the medication as I just mentioned and there may be side effects and the side effects may be worse.”*  *“I am sure once the medication is out, the population is informed about it, they will take it. Yep.”* |
| **Barriers to PrEP** | - Would there be any stigma regarding taking PrEP medications? - Are there any factors that might influence/affect PrEP use in the Haitian community? - What are your thoughts on HIV testing in the Haitian community?? | *"They will think that he/she is someone who is sick."*  *“…that you're promiscuous, you have multiple partners. Mm-hmm. Mm-hmm.”*  *“…the stereotypes of, oh, if you're homo that's why you're taking it, or you with a person that has or been with another man or things like that, that's the...”*  *“And I realize that, I feel like from what I remember seeing that I think they target a specific group. These commercials. And I think that’s a mistake. Because in the commercials that I see with people that are saying “I am taking so and so to prevent…” it’s mostly homosexual males. With that being said, people are going to feel like, ohh that’s for that group. And they’re not going to take it, not even realizing that HIV is for everybody. Everyone gets infected with HIV, you understand. So, I think it’s a mistake on their part targeting just that particular group.”*  *“So is the doctor really giving it to you because that's what's best for you, or because that's what's best for his business?”*    *“We noticed especially in the Haitian community, I am coming back to this, if someone is not leaving the country or if someone is not obligated to be tested, you will never see that person get and AIDS test”*  *“So, yeah, I believe it's very big in within the, the, um, the Haitian community. It's just the fact that people don't want to get checked out. Mm.*  *“A lot of people just don't get tested because they figure what I don't know won't hurt me.”* |
| **Role of church and PrEP** | - How can churches be helpful in promoting PrEP? - Do you think that there would be a role for the church in providing PrEP? | *“I think as a church we can do the same thing by not only just promoting abstinence but realizing that there are people who are not practicing abstinence and for us to be real and knowing that it's a true factor. Like there, there are people that are not practicing abstinence and they're doing it in secret, but as long as if they're doing it, let's make sure they're protecting themselves.”*  *“Um, the same as we do the Health Fairs, having the Health Department come out doing… I mean HIV testing in the mobile, mobile, those big mobile places, I forgot how you call them. Humm, and so that people can be confident…”*  *“I think that's going to be hard to do in this setting because, again, that would be promoting sex. And the church does not promote sex.”*  *“…one of the things that the churches, they might need to do, especially the Haitian churches, they might need to learn, start assessing or see what the people know about HIV, what they think about it…”* |
| **Current services Provided** | - At your church, do members receive any information regarding health or health services? If so, what kinds of services are provided? | *“I would say the focus is more so on reproductive health more than sexual health.”*  *"About three months ago we did a study with a doctor who came to talk with the kids about the dangers of having sex."*  *“… last weekend we had a health, um, fair, um, um, um, inviting the neighborhood. You know, people came through, they had their blood, the pressures checked. Um, I think that they do blood sugar. Yeah, blood sugar. Blood sugar. And so, it was a big thing. So, breast cancer, um, month is big here. “*  *“Um, well, mostly when we do screenings, um, it would be about, um, um, diabetes, hypertension, um, things as far as, um, you screen them for breast cancer, teaching them how to do those, those are the, you know, typical screening that we do. Yeah”* |
| **Interventions to help with PrEP** | - How can PrEP best be promoted? | *"These popular Haitian figures in the community that many people listen to, like my mom used to listen to um, oh gosh, I forgot his name on the radio. Piman bouk. There are other popular Haitian people in the community, and they can also, talk about it, and they have stuff on their radio stations and stuff like that, they can talk about it…”* |
