# Supplementary material for: Bon bagay (good stuff): A faith-based outlook on biomedical prevention among Haitians and Haitian Americans
Source: PLoS One. 2025 Aug 25;20(8):e0330852. doi: 10.1371/journal.pone.0330852 (PMC12377581; doi:10.1371/journal.pone.0330852)
Supplement: Attachment — Submitted filename: PONE-D-25-27087 REV.pdf [file pone.0330852.s003.pdf]

This is an interesting article and could be improved if the following points were clarified.

1. ***Abstract***

   The type of study and design must be indicated.

2. ***Ethics Statement***

   Why wasn't a signed informed consent form used for the whole study? It's striking that the patients gave verbal consent to participate in the research but didn't sign any documents.

3. ***Methods***

   Recruitment

   It is not indicated how the sample size was established.

   Privacy and access to research data

   It is not indicated how privacy and access to research data were protected. This should be mentioned.

   Data Analysis

   The current description of the data analysis process is insufficient for a qualitative study, as it lacks critical details necessary to assess methodological rigor and transparency. Specifically, the manuscript does not indicate the type of qualitative analysis conducted (e.g., thematic analysis, grounded theory), whether an inductive, deductive, or mixed coding approach was used (the summary indicates that it was inductive), or how discrepancies between coders were resolved. The process of codebook development is briefly mentioned, but no information is provided regarding its structure, validation, or use across the dataset. Moreover, the manuscript does not address how data saturation was assessed or what strategies were employed to ensure trustworthiness (e.g., triangulation, reflexivity, audit trail, or member checking). It is recommended that the authors expand this section by clearly outlining the analytic approach, coding procedures, software use, criteria for saturation, and strategies to enhance the credibility of findings.

4. ***Results***

   Although six major themes or categories of analysis were explored, this section does not indicate whether there were emerging categories, as this information is also relevant as it

provides new insights that enrich the study, especially since a semi-structured approach was used for the focus group.

5. Discussion

This is a well-done section, but I think it would be improved if the importance of cultural relevance in health interventions were explored in more depth. This allows for better shaping of health strategies to achieve better outcomes for people's health and well-being, as well as helping to narrow the gaps in access to health information and healthcare.
